# Perceptions of Automatic External Defibrillator Use and Accessibility in the Deaf and Hard-of-Hearing Populations of the United States

**DOI:** 10.7759/cureus.74990

**Published:** 2024-12-02

**Authors:** Erika J Yee, Matthew Kern, Chiu-Hsieh Hsu, Natasha A Moini, Ashley Ungor, Ryan H Yee, Max Klahr, Karl Kern, Daniel L Beskind

**Affiliations:** 1 Emergency Medicine, University of Arizona College of Medicine - Tucson, Tucson, USA; 2 Internal Medicine, Vanderbilt University, Nashville, USA; 3 Biostatistics and Epidemiology, University of Arizona, Tucson, USA; 4 Emergency Medicine, University of Arizona, Tucson, USA; 5 Emergency Medicine, Kaweah Delta Health Care District, Tucson, USA

**Keywords:** automated external defibrillator (aed), basic life support (bls), cardiopulmonary resuscitation, deaf, hard-of-hearing, out-of-hospital cardiac arrest

## Abstract

Background

The use of automatic external defibrillators (AEDs) by lay rescuers can reduce the time to defibrillation and improve survival in out-of-hospital cardiac arrest (OHCA). AEDs use voice prompts to guide users through the defibrillation process, creating a potential barrier for deaf and hard-of-hearing (HoH) individuals. The objective of this study is to assess familiarity with and concerns regarding AED use among members of these communities.

Methods

A 19-question Qualtrics survey was distributed to adults in the United States who self-identified as deaf or HoH. The questions included seven demographics, eight yes/no/unsure, three Likert scales, and one multiple-response question. Quantitative analysis was performed using 95% confidence intervals to compare familiarity with and concerns about AED use among deaf, HoH, and combined groups of respondents.

Results

Of the responses, 500 met the inclusion criteria; 130 (26%) self-identified as deaf, and 370 (74%) self-identified as HoH. Around 460 (92%) were in the 18-40 age group. AED recognition was high among both deaf (90.77%) and HoH (84.59%) respondents, though deaf respondents were less likely than HoH respondents to have seen an AED in a public place (p=0.03) or know how to safely use one (p=0.001). In both the deaf and HoH groups, the top concern regarding AED use was that AEDs were too technical or complicated (61.86% and 56.8%). Of all respondents, 36.4% reported that AEDs were not user-friendly (p=0.034). All participants identified some concerns regarding AED use in emergencies. In addition, 56.15% of deaf and 39.19% of HoH respondents were concerned that AED use is potentially dangerous (p<0.001). There was no statistically significant difference between the two groups in knowing when to use an AED or where to find more information about AEDs.

Conclusion

Deaf and HoH people have specific concerns about AEDs, including the safety and complexity of operating an AED and the accessibility (user-friendliness). In this study, the deaf population is less familiar with using an AED than the HoH population. Possible interventions to address concerns of the deaf and HoH communities include AED training given in American Sign Language (ASL) and updating AED designs with improved visual and non-verbal directions.

## Introduction

Approximately 11 million individuals in the United States identify as deaf or as experiencing significant difficulty hearing [1]. Because automatic external defibrillators (AEDs) use voice prompts and are a necessary part of resuscitation efforts in out-of-hospital cardiac arrest (OHCA), this could create a barrier for their effective use in the deaf and HoH communities. Of the approximately 356,000 people who experience OHCA in the United States annually, less than 10% will survive to hospital discharge [2]. Numerous studies have demonstrated the significant benefit of bystander AED use in cases of OHCA, prompting widespread basic life support (BLS) and AED training [3-6]. Though BLS training and the public availability of AEDs have become more widespread, barriers persist for the deaf and hard-of-hearing (HoH) populations. Examples of these barriers include the delay in calling 911 via telecommunications relay services (text telephones), the reliance of AEDs on voice-prompt guidance, and the scarcity of BLS classes offered in sign language. Together, these challenges can reduce the number of potential rescuers in emergencies and perpetuate a significant disparity in health literacy for the deaf and HoH populations.

Though many studies have investigated the ability of the general, abled population to perform BLS maneuvers, little has been done to evaluate the specific concerns and perceptions of AEDs among the deaf and HoH populations [7-9]. Better understanding the concerns of the deaf and HoH communities regarding AEDs is an important first step in addressing the gap in health literacy and barriers to accessible resources. To our knowledge, this is the first study to assess familiarity with and concerns regarding AED use among members of the deaf and HoH communities. 

## Materials and methods

**Figure 1:**
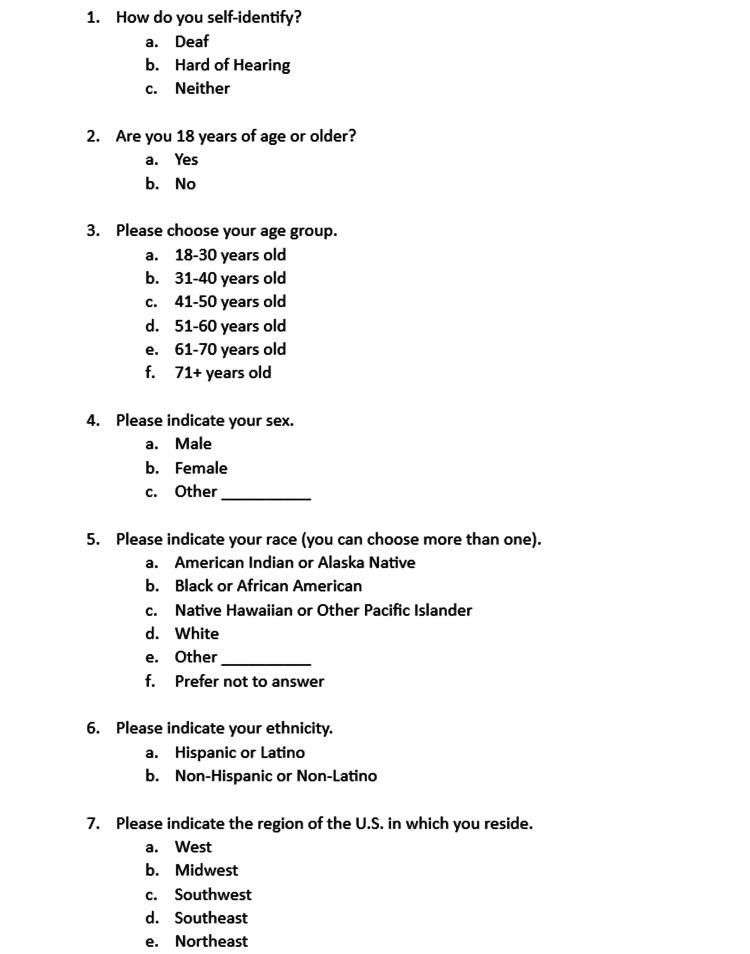
Survey questions

**Figure 2:**
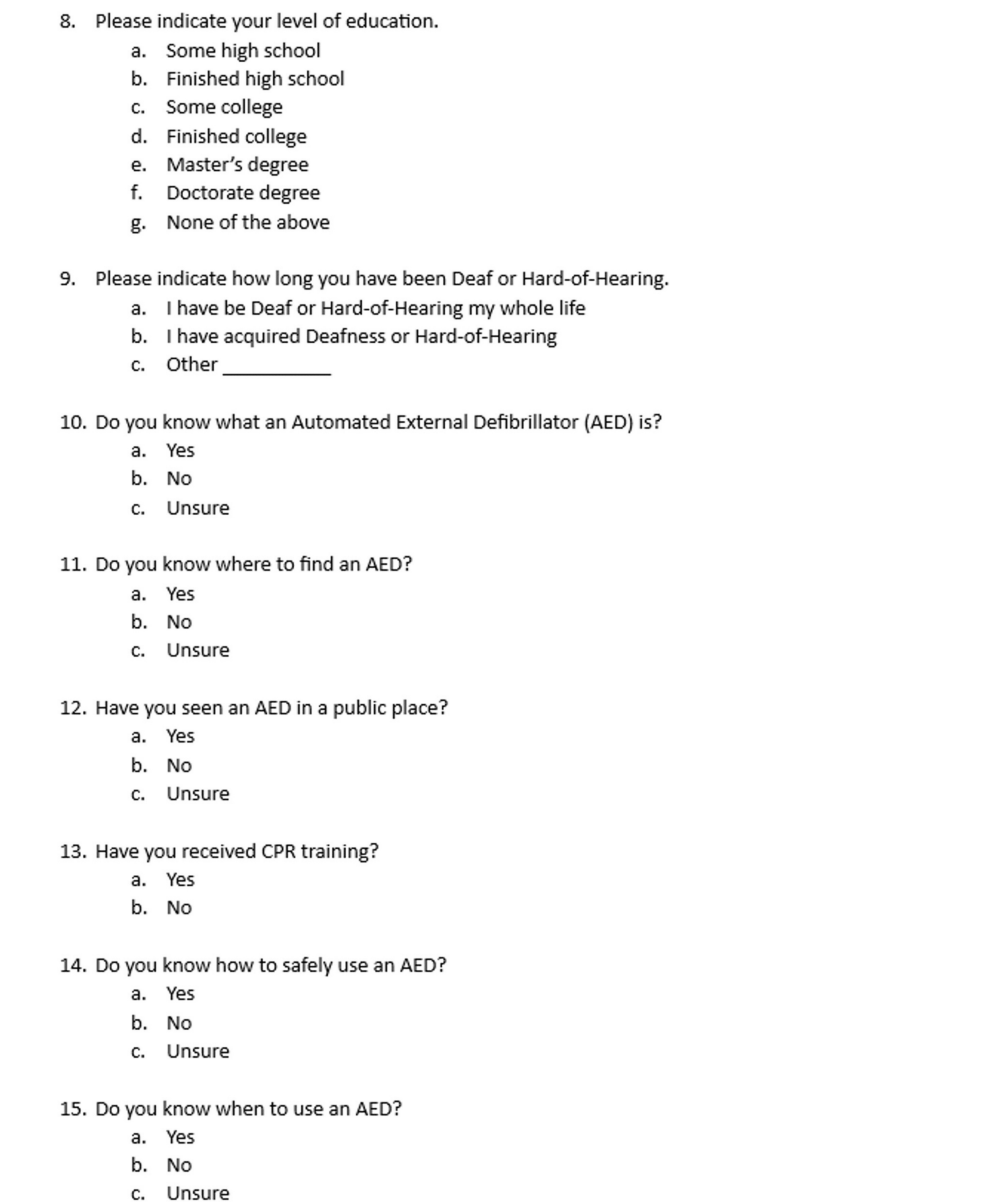
Survey questions AED: automatic external defibrillator; CPR: cardiopulmonary resuscitation

**Figure 3:**
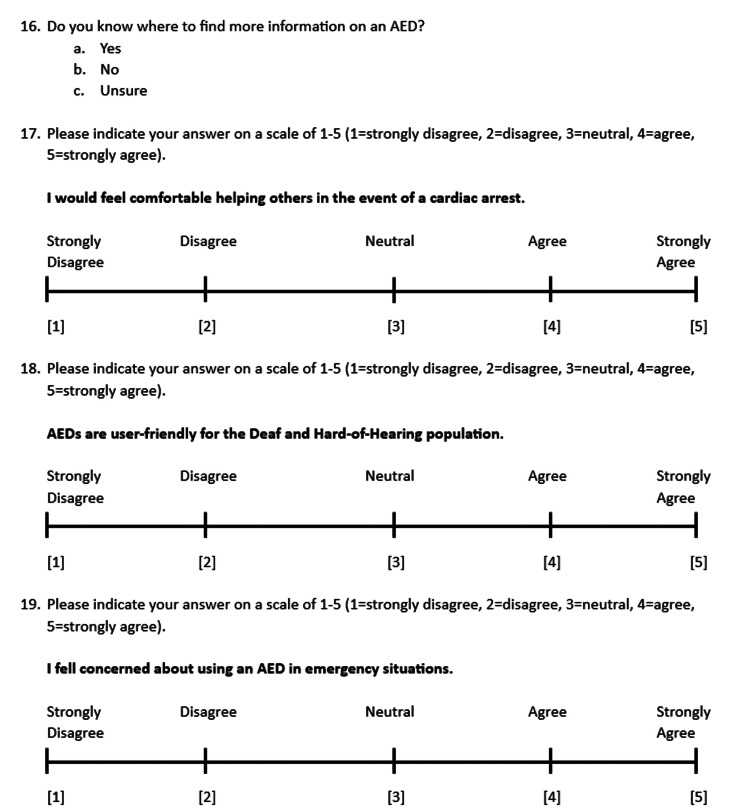
Survey and Likert scale questions AED: automatic external defibrillator

**Figure 4:**
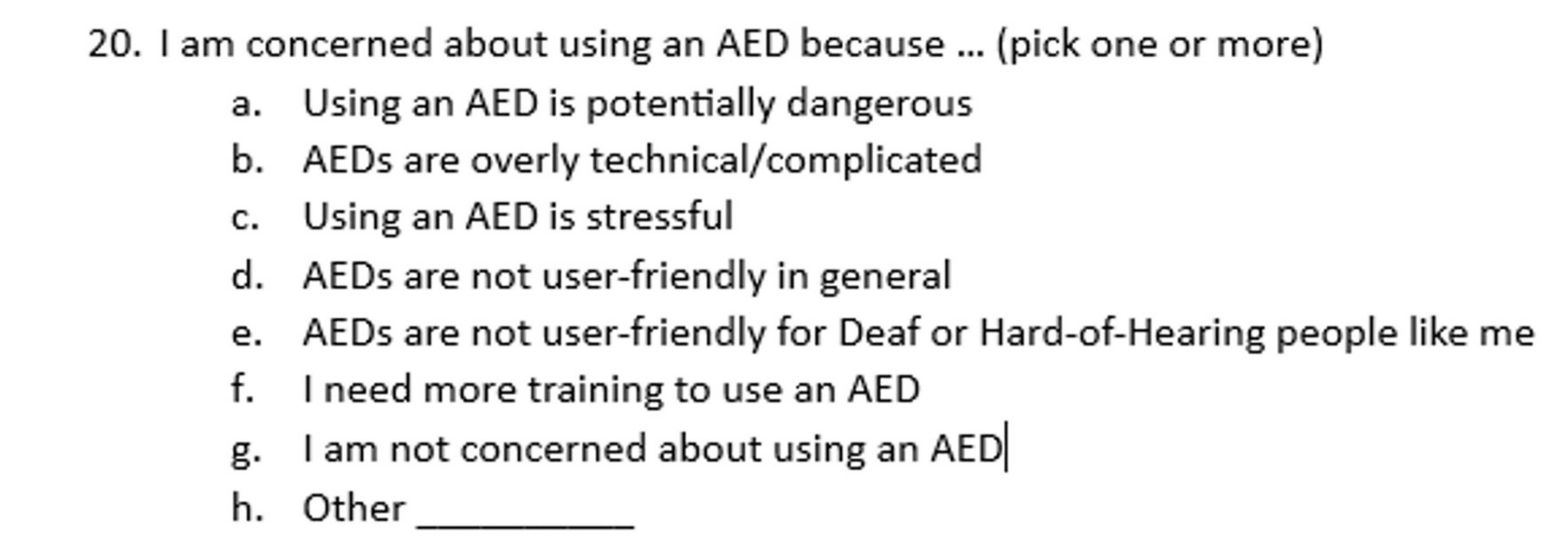
Survey questions AED: automatic external defibrillator

The study was conducted at University of Arizona College of Medicine in Tucson, Arizona. This study was approved by the University of Arizona Institutional Review Board (IRB approval ID 2103565368). Informed consent was obtained from 500 adult participants who self-identified as deaf or HoH. The online survey consisted of demographic questions followed by AED survey questions and was written in English. Figures 1, 2, 3, 4 showcase the complete survey tool. Data was de-identified prior to statistical analysis and kept in a secure, Health Insurance Portability and Accountability Act (HIPAA)-approved online database (University of Arizona Box).

Study design

A 20-question survey was formulated using the online survey tool Qualtrics. Complete survey tools are available in Figures 1, 2, 3, 4. 

The inclusion criteria for the study were participants 18 years or older within the United States who self-identified as deaf or hard-of-hearing. Participants were excluded if they did not meet the above criteria or if their responses were incomplete. 

A link to the online survey was provided to the Arizona Commission for the deaf and hard-of-hearing, which shared the link and QR code on social media platforms as well as email listservs. The survey link and QR code were also shared via the University of Arizona Sarver Heart Center social media platforms and, with permission, on the “Tucson Deaf News” Facebook page (Meta Platforms, Inc., Menlo Park, California, United States). Though the email was distributed primarily to Arizona organizations, the information was shared with other deaf and hard-of-hearing groups in various states. Participants voluntarily entered their emails to enter for a chance to win one of several $50 gift cards. 

Statistical analysis

Participants’ characteristics and responses (all categorical) were summarized using frequency (%) for all participants and by hearing status (deaf and HoH). Logistic regression was performed to identify variables associated with baseline AED knowledge, attitudes toward using an AED, and concerns regarding AEDs, respectively, for all participants and by hearing status. Variables with a p-value <0.05 in the univariate logistic regression analysis were included in the adjusted logistic regression analyses. All statistical analyses were performed using R 4.1.0 (R Foundation for Statistical Computing, Vienna, Austria, https://www.R-project.org/) with the significance level set at 5%. 

## Results

Sample

Participants self-identified their deaf or hard-of-hearing status in their responses. A total of 500 responses met the inclusion criteria. Around 26% (n=130) self-identified as deaf and 74% (n=370) self-identified as HoH. The majority of participants were HoH (74%, n=370), between the ages of 18 and 40 (92%, n=460), White individuals (85%, n=425), non-Hispanic individuals (76%, n=380), male (64%, n=322), residing in the Southwest and Midwest (60.15%), and had completed college (77%, n=384). A summary of study demographics can be found in Table 1. 

**Table 1:** Demographics HoH: hard-of-hearing

Variable		Overall (n=500)	Deaf (n=130)	HoH (n=370)
Age				
	<40 YO	92.00% (n=460)	93.85% (n=122)	91.35% (n=338)
	≥41 YO	8.00% (n=40)	6.15% (n=8)	8.65% % (n=32)
Gender				
	Female	33.80% (n=169)	25.38% (n=33)	36.76% (n=136)
	Male	64.40% (n=322)	67.69% (n=88)	63.24% (n=234)
	Other, unspecified	1.80% (n=9)	6.92% (n=9)	0.00% (n=0)
Education				
	Some high school	4.40% (n=22)	9.23% (n=12)	2.70% (n=10)
	Finished high school	5.00% (n=25)	4.62% (n=6)	5.14% (n=19)
	Some college	13.00% (n=65)	9.23% (n=12)	14.32% (n=53)
	Finished college	34.60% (n=173)	42.31% (n=55)	31.89% (n=118)
	Master’s degree	30.60% (n=153)	33.08% (n=43)	29.73% (n=110)
	Doctorate degree	11.60% (n=58)	1.54% (n=2)	15.14% (n=56)
	None of the above	0.80% (n=4)	0.00% (n=0)	1.08% (n=4)
Race				
	American Indian or Alaska Native	5.80% (n=29)	8.46% (n=11)	4.86% (18)
	Asian	1.40% (n=7)	0.77% (n=1)	1.62% (n=6)
	Black or African American	2.80% (n=14)	4.62% (n=6)	2.16% (n=8)
	Native Hawaiian or Other Pacific Islander	0.60% (n=3)	1.54% (n=2)	0.27% (n=1)
	White	85.00% (n=425)	76.92% (n=100)	87.84% (n=325)
	Multiracial			
	American Indian or Alaskan Native, White	2.40% (n=12)	7.69% (n=10)	0.54% (n=2)
	Asian, White	2.00% (n=10)	0.00% (n=0)	2.70% (n=10)
Ethnicity				
	Hispanic or Latino	24.00% (n=120)	23.85% (n=31)	24.05% (2=89)
	Non-Hispanic or Non-Latino	76.00% (n=380)	76.15% (n=99)	75.95% (n=281)
Region				
	Midwest	24.64% (n=102)	23.15% (n=25)	25.16% (n=77)
	Northeast	13.04% (n=54)	7.41% (n=8)	15.03% (n=46)
	Southeast	11.59% (n=48)	7.41% (n=8)	13.07% (n=40)
	Southwest	35.51% (n=147)	44.44% (n=48)	32.35% (n=99)
	West	15.22% (n=63)	17.59% (n=19)	14.38% (n=44)
	Missing	17.20% (n=86)	16.92% (n=22)	17.29% (n=64)

Survey results

Overall, the majority of participants know what an AED is (n=431 (86.20%)), know where to find an AED (n=383 (76.6%)), know how to safely use an AED (n=336 (67.2%)), have seen an AED in a public place (n=346 (69.62%)), know when to use an AED (n=347 (69.4%)), and know where to find more information about AEDs (n=344 (68.8%)). AED recognition was higher among deaf respondents compared to HoH respondents (90.77% vs. 84.59%). The HoH group had a higher rate of having seen an AED in public (n=266 (72.48%)), knowing when to use an AED (n=247 (69.4%)), knowing where to find an AED (n=296 (80%)), and knowing where to find information about AEDs (n=258 (69.73%)). Around 82.9% of the total group received cardiopulmonary resuscitation (CPR) training, with high rates of training seen in each participant group: (deaf n=100 (77.52%), HoH n=312 (84.78%)).

Half of all participants agreed or strongly agreed with the statement, “I feel comfortable helping others in a cardiac arrest” (n=253 (50.6%), 52.16% HoH vs. 46.15% deaf). Just over half of all participants agreed or strongly agreed that AEDs were user-friendly overall (n=272 (54.4%)).

All 500 participants of the study indicated concerns regarding the use of AEDs (Table 2 and Figure 4, question 20). Of the concerns listed, 54% (n=270) of participants chose “AEDs are overly technical and complicated,” 24% (n=118) chose “I need more training,” 49% (n=245) chose “using an AED is stressful,” 44% (n=218) chose “AEDs are potentially dangerous,” 40% (n=200) chose “AEDs are not user friendly in general,” and 36% (n=182) chose “AEDs are not user friendly for people like me (deaf or HoH).”

**Table 2:** Participant concerns about using an AED AED: automatic external defibrillator; HoH: hard-of-hearing

	Overall (n=500)	Deaf (n=130)	HoH (n=370)	p-value (<0.05)
Need more training	23.60% (n=118)	21.54% (n=28)	24.32% (n=90)	0.520
Using an AED would be stressful	49.00% (n=245)	44.62% (n=58)	50.54% (n=187)	0.245
Using an AED is potentially dangerous	43.60% (n=218)	56.15% (n=73)	39.19% (n=145)	<0.001
AEDs are not generally user-friendly	40.00% (n=200)	28.46% (n=37)	44.05% (n=163)	0.441
AEDs are not user-friendly for deaf/HoH	36.40% (n=182)	29.23% (n=38)	38.92% (n=144)	0.034
AEDs are too technical/complicated	54.00% (n=270)	57.69% (n=75)	52.70% (n=195)	0.326

In the deaf group regarding the concerns listed above, 21.54% (n=28) expressed the need for more training, 44.62% (n=58) worried that using an AED would be stressful, 56.15% (n=73) were concerned that AEDs are potentially dangerous, 28.46% (n=37) felt that AEDs are generally not user-friendly, 29.23% (n=38) felt that AEDs are not user-friendly for deaf or HoH people, and 57.69% (n=75) were concerned that AEDs are too technical or complicated. 

Comparatively, in the HoH group, 24.32% (n=90) expressed the need for more training, 50.54% (n=187) worried that using an AED would be stressful, 39.19% (n=145) felt that an AED is potentially dangerous, 44.05% (n=163) felt that AEDs were generally not user-friendly, 38.92% (n=144) felt that AEDs are not user-friendly for deaf or HoH individuals, and 52.7% (n=195) were concerned that AEDs are too technical or complicated. 

The rates of both AED recognition (90.77% deaf, 84.59% HoH; p=0.082) and CPR training (77.69% deaf, 84.86% HoH; p=0.061) were similar across deaf and HoH participants, although this was not statistically significant. A higher proportion of HoH respondents reported knowing where to find an AED (66.92% deaf, 80% HoH; p=0.03). Similarly, more HoH participants reported seeing AEDs in public places, knowing how to operate an AED safely, and agreeing or strongly agreeing that AEDs are user-friendly for deaf/HoH individuals (on a Likert Scale) (p=0.03; p=0.001; p=0.004). However, HoH individuals were concerned that AEDs are not user-friendly for deaf or HoH individuals (38.92% HoH, 29.23% Deaf; p=0.034) (Table 2). A higher rate of deaf individuals were concerned that using an AED is potentially dangerous (p <0.001). 

Additional subgroup analysis revealed that fewer participants who identified as Hispanic participants reported receiving CPR training (67.50% Hispanic participants; 87.89% non-Hispanic participants; p=0.001). Similarly, fewer Hispanic participants reported having basic knowledge of AEDs (70% Hispanic participants; 91.32% non-Hispanic participants; p=<0.001), knowing where to find an AED (55.83% Hispanic participants; 83.16% non-Hispanic participants; p <0.001), having seen an AED in public places (55.83% Hispanic participants; 74.21% non-Hispanic participants; p=0.01), and knowing how to safely use an AED (45% Hispanic participants; 74.21% non-Hispanic participants; p=0.002). Additionally, respondents without graduate degrees were less likely to know where to find an AED (68.51% no graduate degree; 87.68% graduate degree; p=0.003), have had CPR training (76.82% no graduate degree; 91.47% graduate degree; p=0.009), know how to safely use an AED (54.67% no graduate degree; 84.36% graduate degree; p=0.002), know where to find more information on AEDs (59.86% no graduate degree; 81.04% graduate degree; p=0.001), or know when to use an AED (60.9% no graduate degree; 81.04% graduate degree; p <0.001). 

## Discussion

In this national survey of deaf and hard-of-hearing adults, participants have specific concerns about using AEDs that may prevent members of these communities from effectively deploying an AED and participating in bystander resuscitation efforts. Both deaf and hard-of-hearing participants associated AEDs with being too technical or complicated, potentially dangerous, and not generally user-friendly. Future studies should consider adding focus groups to better understand why both the deaf and HoH groups have these perceptions. Overall, approximately half of the participants did not feel comfortable helping in cardiac arrest scenarios.

Barriers to AED use in the general population for cases of OHCA have been studied [10,11]. These studies identified that despite basic knowledge of AEDs being high in the general population, individuals are concerned with harming patients, not possessing adequate knowledge for AED use, and being unable to perform resuscitation techniques properly. Similarly, a study in Taiwan found the primary concern of using an AED in emergencies was the legal ramifications following AED use [10]. The fear of legal liability associated with AED use has been highlighted in several studies, though participants in this study did not specifically outline this concern [11,12]. 

Barriers to AED use in populations with disabilities have similarly been studied; however, the fears and barriers differ from those of the general population in several ways. Unnikrishnan et al. identified challenges of initiating emergency medical service (EMS) contact and the inability to follow voice prompts of AEDs in individuals who identified as HoH [13]. Despite these challenges, participants in the study performed BLS maneuvers adequately with the presence of a sign language interpreter. Similarly, a study of deaf individuals demonstrated that without training, deaf lay responders were able to utilize AEDs properly with visual AED prompts, and additional training in this population could reduce the time to defibrillation [14]. While these studies evaluate challenges to performing BLS maneuvers with AEDs, few studies have addressed the concerns of AED use in the deaf and HoH populations. To our knowledge, this is the first national study investigating the concerns of the deaf and HoH population exclusively.

In this study, deaf and HoH Hispanic participants were less likely to have a baseline knowledge of AEDs, have CPR training, or know how to use an AED safely. Interestingly, a low rate of Hispanic participants in both the HoH and deaf participant populations felt the need for additional training. A lack of a graduate degree was also associated with a decreased knowledge of how to safely use an AED or when to use one in both the deaf and HoH populations. It is unclear if these trends in this study are generalizable to the general US population, but it would make for a good future study. In addition, a statistically significant higher rate of the deaf population was concerned that AED use could be potentially dangerous, and we are not sure why this is the case, but it should be explored in future studies.

Our study suggests the need for BLS and AED training that is more accessible to the deaf and HoH populations and the need to update existing designs of AEDs with improved visual or non-verbal prompts. The adaptation of AED designs that incorporate visual prompts, closed captions, non-verbal cues, and BLS/AED training with sign language interpreters will improve access to life-saving materials in the deaf and HoH populations. Future studies should explore the impact of device modifications on AED use in the deaf and HoH populations.

Limitations

This survey study has several limitations. Firstly, significantly more participants self-identified as HoH rather than deaf. The majority of participants were White and non-Hispanic individuals. However, the sample still represents deaf and HoH populations that are diverse in race, ethnicity, gender, and education level, and the survey responses came from a variety of regions in the United States. Nevertheless, the generalizability to the US population may be limited. Secondly, self-identification of hearing status by participants may be subjective data rather than an official diagnosis of deafness or hard-of-hearing. Thirdly, the online nature of the study could not prevent duplicate and robotic (bot) responses. However, several actions were taken in the Qualtrics survey to prevent bot responses, including reCAPTCHA, prevention of indexing the survey in search engines, and exclusion of repeat responses from final data analysis. Only participants who met the inclusion criteria and those with access to the link or QR code were able to access the survey study. Channels used to recruit participants may further limit the broad application of the results as they may better reflect the population of the Southwest and Midwest US. This study survey is a convenience survey, which inherently makes the results not representative of the whole deaf or HoH population in the US because of an inability to randomly sample and the subjective nature of the questions. Lastly, the language of the survey was in English, and the phrasing of the survey questions could be interpreted differently if English is not the participant's first language. However, to prevent leading questions, the survey was written and checked using survey methodology by Groves et al. [15]. 

One other interesting finding was the percentage of deaf and HoH in this survey who had prior CPR training was very high (82.9%). This is higher than the national average of 65% in the American population [16]. We are not sure why this is the case and whether there is an emphasis on CPR training in these populations or whether it means this could represent duplicate answers in our online survey, although we took measures to prevent this. 

## Conclusions

In an online survey of deaf and hard-of-hearing adults in the United States, the participants who identified as deaf were significantly less likely to know where to find an AED, safely use an AED, and identify an AED in a public place compared to those who self-identified as HoH. Deaf participants were more concerned about the potential danger associated with AED use. HoH participants felt that AEDs were not generally user-friendly or user-friendly for deaf or HoH individuals. The majority of both deaf and HoH populations indicated that AEDs are too technical or complicated. Developing AEDs that are more user-friendly for the deaf and HoH populations and providing flexible, adaptable, and accessible BLS and AED training for deaf or hard-of-hearing individuals may improve OHCA recognition and increase AED use in OHCA in these populations.
